# Combinatorial Treatment with Apelin-13 Enhances the Therapeutic Efficacy of a Preconditioned Cell-Based Therapy for Peripheral Ischemia

**DOI:** 10.1038/srep19379

**Published:** 2016-01-14

**Authors:** Makoto Samura, Noriyasu Morikage, Kotaro Suehiro, Yuya Tanaka, Tamami Nakamura, Arata Nishimoto, Koji Ueno, Tohru Hosoyama, Kimikazu Hamano

**Affiliations:** 1 Department of Surgery and Clinical Science, Yamaguchi University Graduate School of Medicine, 1-1-1 Minamikogushi, Ube, Yamaguchi 755-0836, Japan; 2 Center for Regenerative Medicine, Yamaguchi University Graduate School of Medicine, 1-1-1 Minamikogushi, Ube, Yamaguchi 755-0836, Japan

## Abstract

Hypoxic pretreatment of peripheral blood mononuclear cells (PBMNCs) enhances therapeutic angiogenesis in ischemic tissues after cell transplantation. However, newly formed vessels generated using this approach are immature and insufficient for promoting functional recovery from severe ischemia. In this study, we examined whether apelin-13, a regulator of vessel maturation, could be an effective promoter of therapeutic angiogenesis, following severe limb ischemia. Combinatorial treatment of hypoxic preconditioned PBMNCs with apelin-13 resulted in increased blood perfusion and vascular reactivity in ischemic mouse hindlimbs compared with a monotherapy comprising each factor. Apelin-13 upregulated expression of PDGF-BB and TGF-β1 in hypoxic PBMNCs, as well as that of PDGFR-β in vascular smooth muscle cells (VSMCs). Proliferation and migration of VSMCs treated with apelin-13 was accelerated in the presence of PDGF-BB. Interestingly, expression of an apelin receptor, APJ, in PBMNC was increased under hypoxia but not under normoxia. In addition, an *in vitro* angiogenesis assay using a co-culture model comprising mouse thoracic aorta, hypoxic PBMNCs, and apelin-13 demonstrated that combinatorial treatment recruited mural cells to sprouted vessel outgrowths from the aortic ring, thereby promoting neovessel maturation. Thus, combinatorial injection of hypoxic PBMNCs and apelin-13 could be an effective therapeutic strategy for patients with severe ischemic diseases.

Cell-based therapy is a promising strategy for induction of therapeutic angiogenesis in ischemic tissues. The mechanisms underlying therapeutic angiogenesis are thought to be either direct or indirect, depending on graft cell types and technical approaches. Peripheral blood mononuclear cells (PBMNCs) are one of most useful cell types for induction of therapeutic angiogenesis, as they can be easily and safely isolated from patients without anesthesia, even in those at a high risk for advanced cardiovascular disease^1^. Soluble factors from transplanted PBMNCs are most likely responsible for the therapeutic effects, including promotion of neovascularization; endothelial progenitor cells (EPCs) present among transplanted PBMNCs could also contribute to therapeutic angiogenesis^2^,^3^,^4^,^5^. However, the clinical benefits of PBMNC transplantation are limited in severe limb ischemia, with therapeutic effects being insufficient to treat patients, similar to that observed with other cell-based therapies^6^,^7^,^8^. Low graft retention and survival in ischemic tissues are common problems that limit the therapeutic effects of cell-based therapies, with the majority of transplanted cells being removed from ischemic tissues within a few days post-transplantation^9^,^10^. Thus, the efficacy of cell-based therapy could be improved if graft cells were retained for longer periods in ischemic tissues. We previously reported that hypoxic pretreatment of graft cells enhances oxidative stress tolerance, cell adhesion capacity, and vascular endothelial cell growth factor (VEGF) secretion of PBMNCs, resulting in acceleration of angiogenesis and improvement of blood flow in ischemic hindlimbs^11^,^12^,^13^,^14^.

An important challenge in cell-based therapy is how to stimulate functional and morphological maturation of neovessels after cell transplantation in ischemic tissues. Newly formed vessels, including capillaries, must be functional after cell transplantation to supply sufficient blood flow to meet the oxygen and metabolic needs of ischemic regions. However, neovessels after cell transplantation is insufficient for ischemic tissues, possibly owing to immature vessel formation^15^. Many monotherapeutic interventions were not effective in large-scale clinical trials of therapeutic angiogenesis in ischemic tissues^16^. A recent study demonstrated that a combinatorial delivery of multiple growth factors resulted in formation of mature vessels in ischemic tissues, with improved perfusion^17^,^18^. In addition, preclinical evidence also supports the notion that combinatorial approaches using a cell-based therapy in parallel with gene therapy induces neovessel maturation and improved hindlimb function^19^. These reports suggest that a combination treatment comprising a growth factor component is a potentially effective approach to enhance cell-based therapeutic angiogenesis in ischemic tissues.

Vessel maturation requires mural cell recruitment, matrix deposition, and vessel wall formation, followed by the sprouting of endothelial cells (ECs)^20^. In sprouting angiogenesis, angiopoietin-1 (Ang-1) and apelin function as important factors that support mature EC sprouting from pre-existing vessels. For example, Ang-1 produced by mural cells regulates EC migration, adhesion, and survival^21^. Apelin, an endogenous ligand for APJ, regulates neovessel caliber size^22^,^23^,^24^. Apelin occurs in two different isoforms, long (apelin-36) and short (apelin-13) peptides; both peptides activate APJ on the surface of ECs to stimulate their assembly and proliferation^25^. Based on their respective roles in vessel maturation, both Ang-1 and apelin are thought to be prospective factors for promotion of therapeutic angiogenesis in ischemic tissues. Indeed, combinatorial delivery of Ang-1 enhanced the therapeutic effects of a cell-based therapy using bone marrow-derived mononuclear cells in rabbit hindlimb ischemia models, and gene delivery of apelin in parallel with VEGF similarly resulted in formation of well-developed vessels in ischemic mouse hindlimbs^19^,^22^. These reports indicate that a strategy targeting vessel maturation is a promising approach to enhance therapeutic angiogenesis.

Here, we propose a novel combination therapy designed to induce vessel maturation, using apelin infusion in parallel with a cell-based pro-angiogenic therapy. In particular, a triple combinatorial strategy comprising hypoxic preconditioning of graft cells, cell transplantation, and apelin-mediated vessel maturation could be an effective method of improving ischemia in peripheral tissues.

## Results

### Combinatorial injection of preconditioned PBMNCs and apelin-13 promotes the development of new functional vessels in ischemic hindlimbs

**Figure 1:**
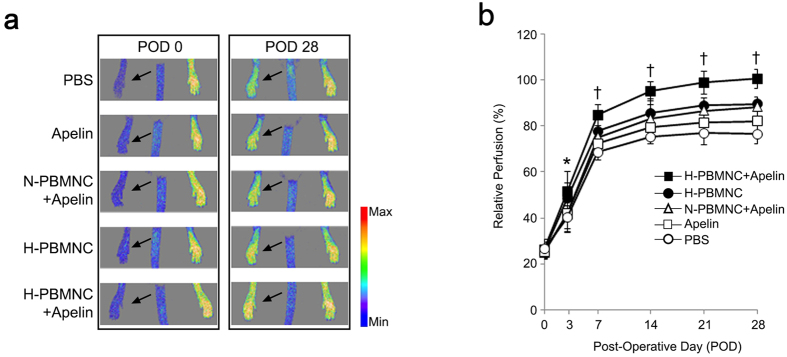
Triple therapeutic strategy using hypoxic preconditioning of graft cells, cell-based pro-angiogenic therapy, and apelin-13 infusion restores blood perfusion in ischemic hindlimbs. Sequential blood perfusion in the ischemic hindlimb after therapeutic treatment was measured by the laser Doppler scanning system. (**a**) Blood perfusion in left legs (arrows) was decreased at post-operative day (POD) 0 and improved at POD 28 in all experimental groups. (**b**) Quantitative analysis of blood perfusion indicates that a combination of preconditioned PBMNC and apelin-13 infusion resulted in significantly higher recovery of blood perfusion in ischemic hindlimbs compared with that of all other groups. PBS; PBS injected group, apelin; apelin-13 injected group, N-PBMNC + apelin; co-injected group of normally cultured PBMNCs and apelin-13, H-PBMNC: hypoxia preconditioned PBMNC-injected group, H-PBMNC + apelin: co-injected group of hypoxia preconditioned PBMNCs and apelin-13. *p < 0.05 *vs*. PBS; ^†^*p* < 0.01 *vs*. other four groups.

To evaluate blood perfusion of ischemic hindlimbs, laser Doppler scanning was serially performed after treatment (Fig. 1a), and the perfusion rate was compared between the five experimental groups. Similar to our previous studies, transplantation of PBMNCs resulted in a greater recovery of blood perfusion compared with that observed in the PBS group. More importantly, blood perfusion from POD 7 to POD 28 was markedly increased in the group receiving preconditioned PBMNCs with apelin-13 (H-PBMNC + apelin) among the PBMNC injection groups; blood perfusion in the H-PBMNC + apelin group recovered to the pre-operative state value at POD 28. There was no significant difference in blood perfusion between the H-PBMNC and the N-PBMNC + apelin groups at POD 28 (Fig. 1b).

**Figure 2:**
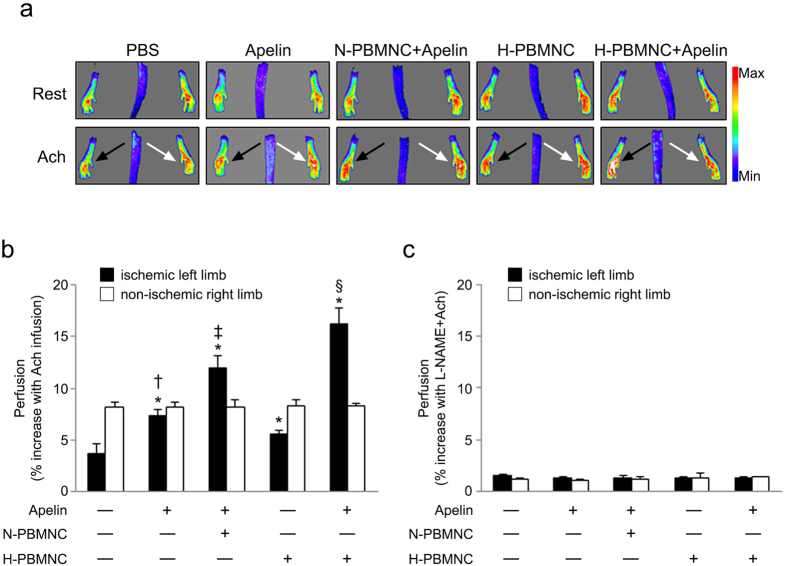
Co-administration of apelin-13 with preconditioned PBMNCs resulted in neovessel with vasodilation potential in ischemic hindlimbs. (**a**) Representative perfusion images before (Rest) and after (Ach) infusion. Acetylcholine (Ach) was intraperitoneally injected into hindlimb ischemia mice at POD 28, and blood perfusion was immediately measured using a laser Doppler scanning system. Black and white arrows indicate ischemic and non-ischemic legs, respectively. (**b**) Quantitative analysis of blood perfusion after Ach infusion in the ischemic (black columns) or non-ischemic (white columns) hindlimbs. Co-injection of apelin-13 with PBMNCs significantly increased blood perfusion in ischemic hindlimbs. (**c**) Quantitative analysis of blood perfusion after Ach and L-NAME infusion in ischemic (black columns) and non-ischemic (white columns) hindlimbs. Vasodilation of neovessels in ischemic hindlimbs was blocked by a nitric oxide inhibitor. *p < 0.01 *vs*. PBS; ^†^*p* < 0.01 *vs*. H-PBMNC, ^‡^*p* < 0.01 *vs*. apelin and H-PBMNC; ^§^*p* < 0.01 *vs*. apelin, apelin + N-PBMNC, and H-PBMNC.

We next examined whether combinatorial injection of PBMNCs and apelin-13 modifies the development of functionally mature vessels in ischemic hindlimbs. We used an Ach stimulation model to evaluate the vasodilation potential of newly formed vessels after treatment^26^. At POD 28, Ach-mediated artery relaxation significantly increased blood perfusion in all treatment groups (Fig. 2a,b). In particular, the H-PBMNC + apelin group showed the strongest reactivity to Ach, suggesting that a combination of preconditioned PBMNCs and apelin-13 induced formation of new functional vessels post-treatment (Fig. 2a). Hindlimb reperfusion followed by Ach infusion was blocked by L-NAME, an inhibitor of nitric oxide, indicating the specificity of Ach-mediated vessel vasodilation in this model (Fig. 1d).

**Figure 3:**
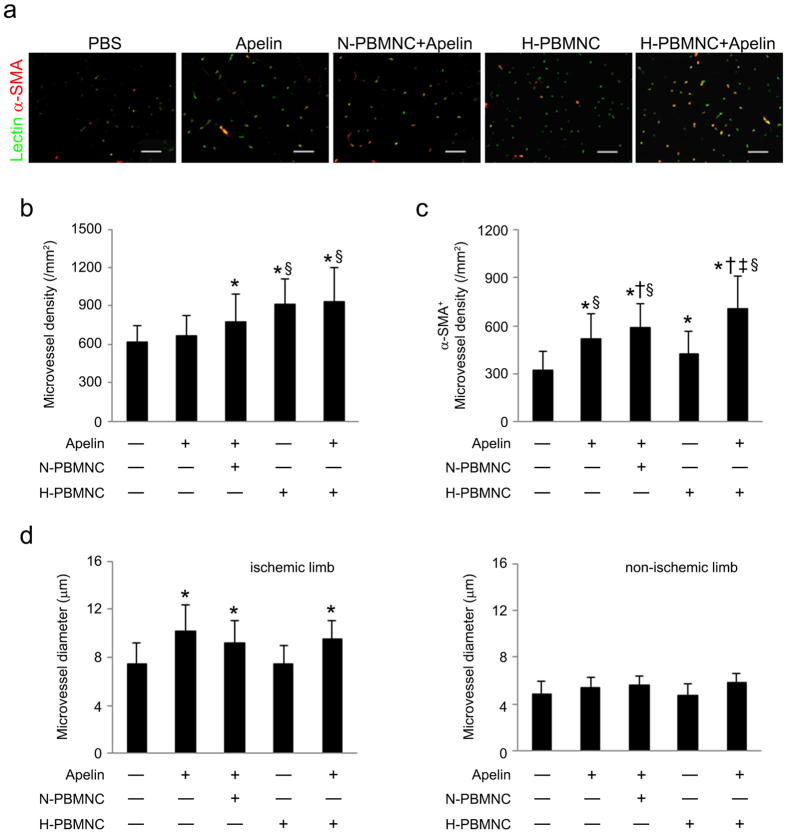
Co-injection of apelin-13 with preconditioned PBMNCs induces development of new mature vessels in ischemic hindlimbs. At POD 28, skeletal muscles were collected from ischemic hindlimbs, and the numbers of mature microvessels were calculated in cross-sections. (**a**) Representative images of microvessels in muscle cross-sections. Endothelial cells and vascular smooth muscle cells were visualized using DyLite488-conjugated Lectin (green) and Cy3-conjugated anti-α-SMA (red) antibodies, respectively. Scale bar = 50 μm. (**b**) Quantitative analysis of total microvessels (Lectin^+^) in ischemic limbs was performed in muscle cross sections. (**c**) Quantitative analysis of α-SMA^+^ microvessel density in ischemic limbs. (**d**) Quantitative analysis of microvessel diameter in ischemic limbs. (**e**) Quantitative analysis of microvessel diameter in non-ischemic limbs. *p < 0.01 *vs*. PBS and apelin; ^†^*p* < 0.01 *vs*. apelin; ^‡^*p* < 0.01 *vs*. apelin + N-PBMNC; ^§^*p* < 0.01 *vs*. H-PBMNC.

Consistent with our previous studies, transplantation of hypoxic preconditioned PBMNCs resulted in new vessel formation in ischemic hindlimbs (Fig. 3a,b). However, a single delivery of preconditioned PBMNCs resulted in smaller vessels compared with those of apelin-13-injected groups, although vessel numbers were still increased; co-administration of apelin-13 and PBMNCs induced the formation of well-developed vessels supported by α-SMA^+^ smooth muscle cells. Notably, preconditioned PBMNCs induced superior new vessel development in cooperation with apelin-13 compared with that of untreated PBMNCs (Fig. 3c,d).

### Hypoxic preconditioning increases PBMNC sensitivity to apelin-13 via upregulation of APJ expression

**Figure 4:**
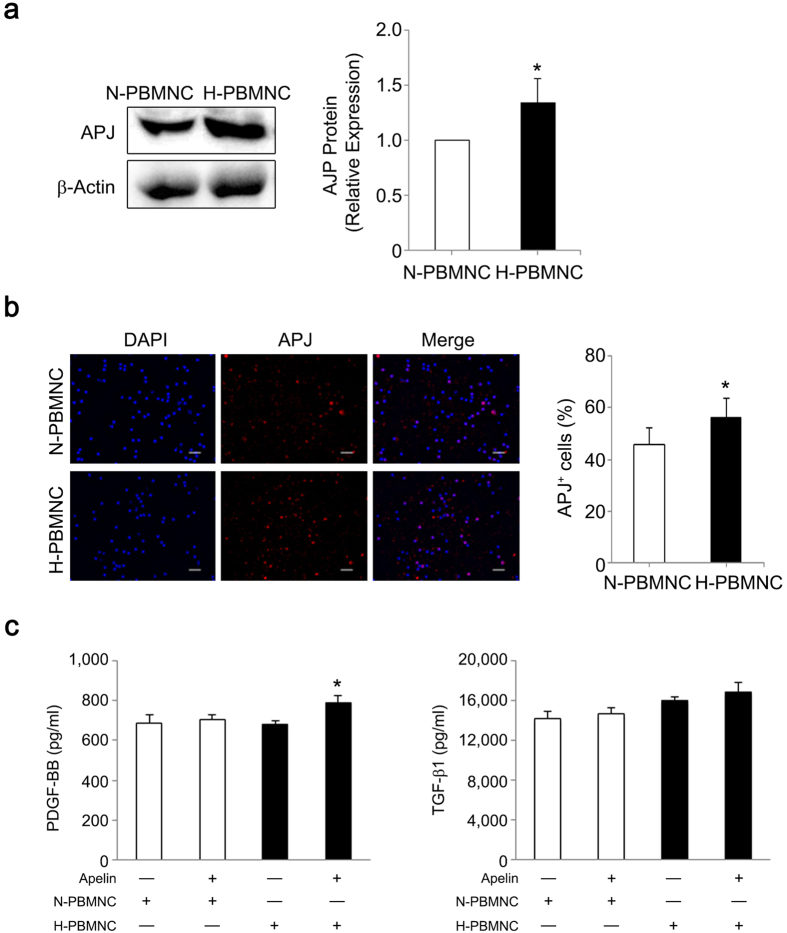
Hypoxic preconditioning increases PBMNC sensitivity to apelin-13 via upregulation of APJ expression, leading to growth factor secretion. (**a**) APJ protein expression was significantly increased in hypoxic PBMNCs. *p < 0.05 *vs*. N-PBMNCs. (**b**) Hypoxic preconditioning increased APJ-expressing PBMNCs. Scale bar = 20 μm. *p < 0.01 *vs*. N-PBMNCs. (**c**) Secretion of PDGF-BB and TGF-β1 was accelerated in preconditioned PBMNCs. Concentrations of PDGF-BB and TGF-β1 in supernatants were analyzed using ELISA. *p < 0.01 *vs*. N-PBMNC group; ^†^*p* < 0.01 *vs*. H-PBMNC.

There is a putative hypoxia response element (HRE) in the first intron of human *APJ*, which encodes an apelin receptor^27^, suggesting that hypoxic preconditioning could enhance PBMNC reactivity to apelin-13 through upregulation of APJ expression. Expression of APJ protein and the numbers of APJ-expressing cells were significantly increased in hypoxic preconditioned mouse PBMNCs (Fig. 4a,b). These results suggest that apelin-13 affects certain cellular functions of preconditioned PBMNCs in addition to promoting vessel maturation. Based on *in vivo* analyses, we hypothesized that apelin-13 upregulates soluble factors associated with mural cell recruitment, especially in preconditioned PBMNCs. Therefore, we examined whether apelin-13 influenced growth factor secretion in preconditioned PBMNCs by modifying APJ expression. We observed that PBMNC-derived secretion of PDGF-BB and TGFβ-1, which play an essential role in mural cell recruitment to nascent blood vessels^28^,^29^, was increased by apelin-13 stimulation and was significantly higher in H-PBMNC than in N-PBMNCs (Fig. 4c). Other soluble factors, including VEGF, bFGF, and HGF, which are critical factors for angiogenesis, were not influenced by the presence of apelin-13 (data not shown). Taken together, a major effect of apelin-13 on PBMNCs was upregulation of factors associated with smooth muscle cell function.

### Ischemia induces APJ expression in VSMCs of arteries and arterioles

**Figure 5:**
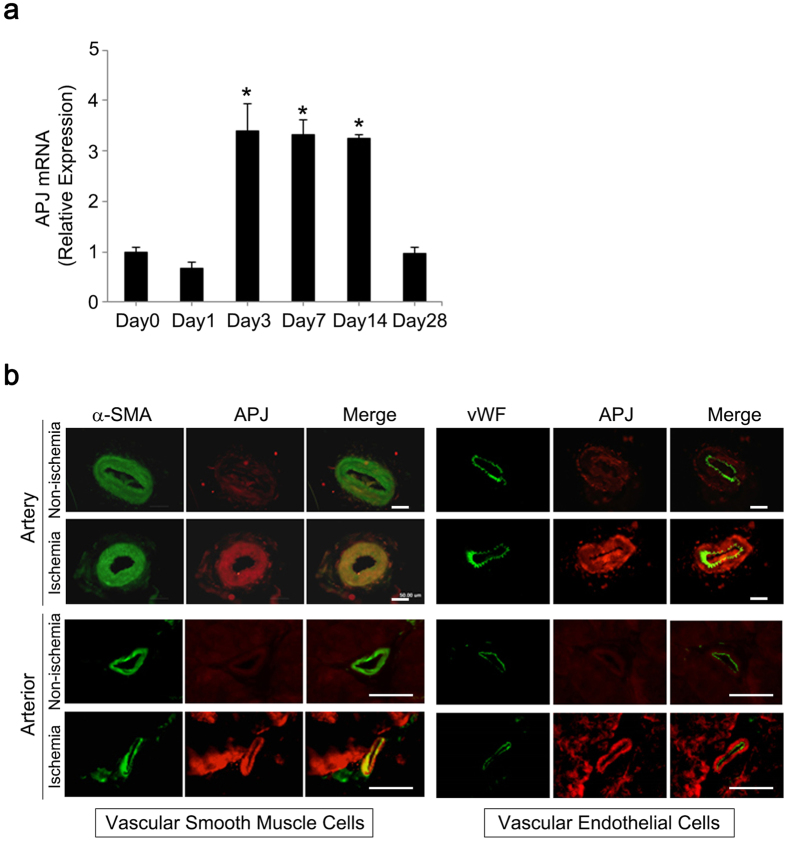
APJ expression in vascular smooth muscle cells of ischemic arteries. (**a**) Quantitative RT-PCR for *APJ* gene expression was performed in ischemic arteries from mouse hindlimbs at day 0 (pre-ligation), 1, 3, 7, 14, and 28 post-ligation. *APJ* mRNA level in ligated arteries was significantly increased between days 3 and 14 post-ligation and returned to baseline level at day 28 post-ligation. *p < 0.01 *vs*. baseline (day 0). (**b**) Vascular smooth muscle cells, but not endothelial cells, expressed *APJ* in ischemic arteries. At day 14, ligated arteries were collected and immunohistochemistry for APJ (red), vWF (green), and α-SMA (green) was performed to identify cells expressing APJ in ischemic arteries. Scale bar = 50 μm.

Apelin-13 binds to the apelin receptor APJ on vascular endothelial cells to induce vascular maturation^22^. APJ is also expressed in VSMCs, and the apelin/APJ axis is associated with vasoconstriction^30^. To identify cellular targets of apelin-13 in ischemic hindlimbs, we evaluated APJ expression in ischemic arteries and arterioles using a femoral artery ligation mouse model. In this model, *APJ* mRNA levels in femoral and saphenous arteries were significantly increased at days 3 and 14 post-ligation (3-fold increase from day 0); expression levels returned to the baseline value at day 28 (Fig. 5a). We next performed a protein expression analysis to identify cells expressing APJ in ischemic arteries at day 14 post-ligation. In arteries and arterioles of ischemic limbs, APJ expression was restricted to VSMCs and was not detected in endothelial cells (ECs). In non-ischemic limbs, APJ was undetectable in both VSMCs and ECs (Fig. 5b).

### Apelin-13 enhances PDGF-BB-induced VSMC proliferation and migration

**Figure 6:**
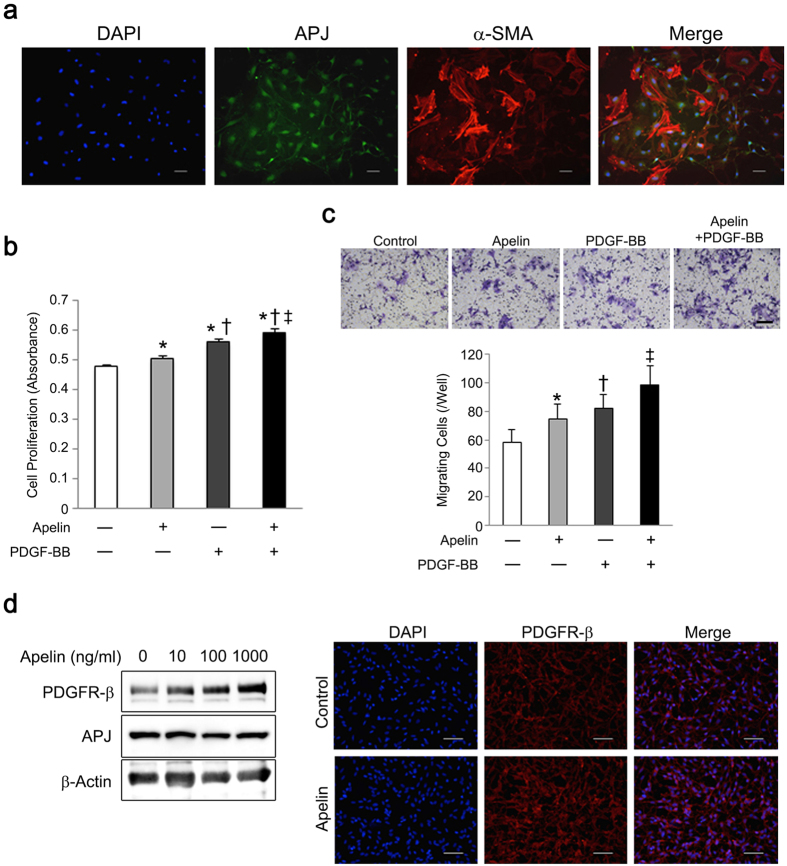
Apelin-13 accelerates proliferation and migration of VSMCs in the presence of PDGF-BB. (**a**) Vascular smooth muscle cells express APJ. Representative images of immunocytochemistry for APJ (green) and α-SMA (red) are shown. Nuclei were stained with DAPI (blue). (**b**) Apelin-13 enhances the effect of PDGF-BB on VSMC proliferation. Apelin-13 accelerated VSMC proliferation as well as PDGF-BB production, and these effects were collaboratively enhanced in a culture containing apelin-13 and PDGF-BB. (**c**) Apelin-13 enhanced the effect of PDGF-BB on VSMC migration. Apelin-13 also enhanced the effect of PDGF-BB on VSMC migration. (**d**) Apelin-13 enhanced PDGFR-β expression in VSMCs. Apelin-13 was added to the VSMC culture and expression of PDGFR-β and APJ was analyzed using western blotting and immunocytochemistry. PDGFR-β expression on VSMCs was dose-dependently increased following apelin-13 administration (left). Immunocytochemistry for PDGFR-β (red) also showed increased PDGFR-β expression in VSMCs after treatment with apelin-13 (1 μM) for 24 h (right). Scale bar = 50 μm. *p < 0.01 *vs*. control; ^†^*p* < 0.01 *vs*. apelin; ^‡^*p* < 0.01 *vs*. PDGF-BB.

Increased APJ expression in VSMCs in ischemic hindlimbs led us to investigate whether apelin-13 influenced VSMC function. We used primary human coronary artery smooth muscle cells to investigate the effect of apelin-13 on proliferation and migration, because of expression of APJ in cultured human VSMCs (Fig. 6a). Apelin-13 accelerated cell proliferation and migration of human VSMCs, and these effects were enhanced when cells were cultured with PDGF-BB (Fig. 6b,c). Thus, apelin-13 is a potential stimulator of VSMC function. Interestingly, apelin-13 also dose-dependently increased PDGF receptor β (PDGFR-β) expression in human VSMCs, suggesting that apelin-13 enhances VSMC sensitivity to PDGF-BB in addition to its direct effects on cell function (Fig. 6d).

### Apelin-13 and preconditioned PBMNCs increase vessel sprouting and maturation of new vessels

**Figure 7:**
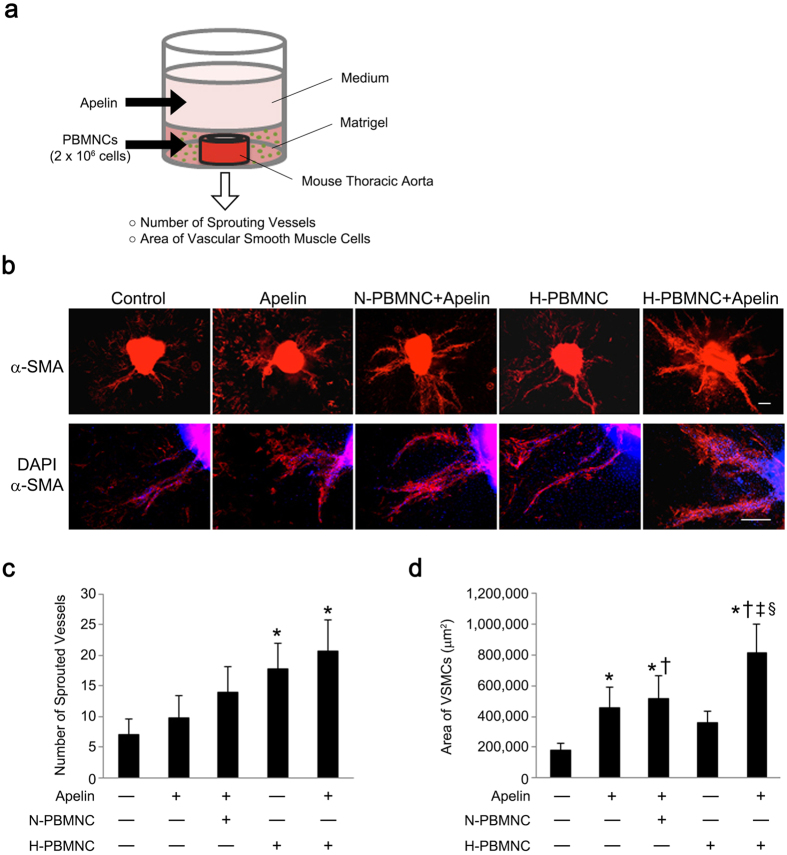
Apelin induces maturation of neovessels in cooperation with preconditioned PBMNCs. (**a**) Scheme of the aortic ring assay using thoracic aorta, PBMNCs, and apelin-13. Mouse thoracic aorta and PBMNCs were embedded in Matrigel, and apelin-13 was added in a layered medium. (**b**) Representative images of the aortic ring and sprouted vessels. Vascular smooth muscle cells surrounding sprouted neovessels were identified by α-SMA expression (red). Scale bar = 200 μm. (**c**) Preconditioned PBMNCs and apelin-13 accelerated sprouting from the aortic ring. The number of sprouted vessels forming the aortic ring was significantly increased in the group of apelin-13 and H-PBMNC. *p < 0.01 *vs*. PBS. (**d**) Combination of apelin-13 and preconditioned PBMNCs enhanced maturation of neovessels sprouted from the aortic ring. The maturation of neovessels was quantitatively estimated by the area of α-SMA^+^ smooth muscle cells in each field. *p < 0.05 *vs*. PBS; ^†^*p* < 0.01 *vs*. apelin; ^‡^*p* < 0.05 *vs*. apelin + N-PBMNC; ^§^*p* < 0.01 *vs*. H-PBMNC.

In experiments using mouse models, we demonstrated that apelin-13 infusion in parallel with preconditioned PBMNCs resulted in increased numbers of well-developed vessels in ischemic hindlimbs. However, it was unclear whether apelin-13 synergistically contributed to the maturation of newly formed vessels following PBMNC transplantation. To investigate potential synergistic effects of apelin-13 and preconditioned PBMNCs on new vessel formation, mouse thoracic aorta and PBMNCs were embedded in Matrigel^®^, and growth medium including apelin-13 was added onto the matrix layer (Fig. 7a). At day 7, the number of sprouted vessels was evaluated. Apelin-13 did not affect sprouting of vessels when combined with untreated PBMNCs (N-PBMNC), although an accumulation of VSMCs to new vessels was increased in all apelin-13 groups (Fig. 7b–d). However, a combination of apelin-13 and H-PBMNC resulted in maturation of newly formed vessels, while H-PBMNC alone induced new vessel formation only (Fig. 7b,d). These results demonstrate the synergistic effects of co-treatment with apelin-13 and preconditioned PBMNCs on angiogenesis.

## Discussion

In this study, we demonstrated that a triple combinatorial strategy using PBMNC transplantation, preconditioned hypoxic graft cells, and apelin-13 infusion is an effective therapeutic approach for severe peripheral ischemia. On studying mouse ischemic models, we found that a combination therapy remarkably increased functional neovessels with NO-dependent vasodilation potential, leading to improvement of blood perfusion in ischemic hindlimbs. Although a single injection of preconditioned PBMNCs induced a significant increase in Ach-responsive vessel vasodilation in ischemic limbs, blood perfusion did not reach the steady state level observed in non-ischemic limbs. This finding suggests that transplantation of preconditioned PBMNCs, a conventional therapeutic approach, results in insufficient recovery in severe ischemic limbs. Notably, α-SMA^+^ vessels in ischemic hindlimbs were significantly increased upon treatment with a combination of apelin-13 and PBMNCs, indicating that apelin-13 increased the therapeutic efficacy of PBMNCs transplantation. Importantly, the combination of apelin-13 and preconditioned PBMNCs showed a greater effect on vessel maturation compared with that of a combination using untreated PBMNCs, although there were no significant differences in vessel numbers between these groups. Taken together, a triple combination approach could be a better strategy for induction of therapeutic angiogenesis in severe hindlimb ischemia. As a similar therapeutic concept, it has been demonstrated that a combination of cell transplantation with *Ang1* gene delivery resulted in both quantitative and qualitative angiogenesis in a rabbit hindlimb ischemia model^19^. Thus, a combination of cell transplantation and delivery of one or more vessel maturation factors could be a promising therapeutic strategy for severe hindlimb ischemia. As another effect, we also found that apelin-13 independently reduced fibrosis in ischemic hindlimbs, indicating an additional therapeutic benefit of combination therapy with apelin-13 (Supplementary Figure S1). In addition, we demonstrated a synergistic effect of apelin-13 with a cell-based therapy, which was most enhanced with addition of H-PBMNCs rather than N-PBMNCs. This suggests that hypoxic preconditioning is essential for induction of superior effects and is a more attractive therapeutic strategy compared with those of conventional cell-based therapies.

Based on *in vitro* studies, apelin-13 has at least two points of action that could enhance the efficacy of therapeutic angiogenesis induced by cell transplantation. One is an effect on preconditioned PBMNC to increase PDGF-BB secretion; the other is an effect on VSMC to enhance PDGFR-β expression. The PDGFR signaling pathway plays a major role in vessel maturation, particularly in the recruitment of mural cells^28^,^31^. Thus, this bidirectional action of apelin-13 involving both PBMNCs and VSMCs could potentially affect VSMC function through the PDGFR signaling pathway, resulting in maturation of newly formed vessels in ischemic hindlimbs. It has been demonstrated that hypoxia upregulates APJ in hepatic stellate cells and hepatocytes, and that activation of the apelin/APJ signaling pathway induces expression of PDGF-BB^32^. These findings indicate a possible mechanism in which increased APJ expression in preconditioned PBMNCs facilitates a cellular responsiveness to apelin-13, consequently increasing PDGF-BB expression. Indeed, PDGF-BB secretion was accelerated only in preconditioned PBMNCs, indicating that APJ upregulation by hypoxia was important for enhancement of neovessel maturation. A similar strategy could be available using preconditioned PBMNCs and angiopoietin-1, as hypoxia stimulates Tie-2 expression, which is an angiopoietin-1 receptor in ECs^33^. However, we demonstrated that apelin-13 acts on VSMC kinetics, consistent with previous studies^30^,^34^. We also found that apelin-13 upregulates PDGFR-β in VSMCs and that co-administration of apelin-13 with PDGF-BB enhances VSMC kinetics. These synergistic effects is convenient to combine the effects of apelin-13 on preconditioned PBMNCs. Upregulation of PDGFR-β by dosed apelin-13 treatment increases VSMC reactivity toward PDGF-BB from preconditioned PBMNCs. In addition, transient upregulation of APJ in VSMCs of ischemic arteries after femoral artery ligation indicates that apelin-13-mediated induction of PDGFR-β was increased in VSMCs in ischemic hindlimbs, resulting in enhanced VSMC proliferation and migration in the triple combinatorial therapy. Thus, a combination therapy comprising preconditioned PBMNCs and apelin-13 resulting in higher therapeutic efficacy in ischemic hindlimbs.

In addition to PBMNC transplantation, apelin has already been tested in Phase I clinical trials to assess its safety as a therapy for cardiovascular diseases^35^,^36^. Apelin-13 instability *in vivo* represents a potential challenge with respect to application of this molecule in clinical settings. However, a recent study demonstrated that nano-liposomal encapsulation stabilized apelin-13, and that administration of this preparation resulted in attenuation of cardiac dysfunction in animal models^37^. Thus, encapsulation in nano-liposomes enables apelin-13 stabilization in the circulatory system, facilitating delivery to target tissues. In conclusion, a combination of PBMNC transplantation and apelin infusion represents a promising therapeutic option for treatment of severe hindlimb ischemia.

## Materials and Methods

### Animals

Male C57BL/6 mice (aged 8–9 weeks) were obtained from a commercial breeder (Japan SLC) and maintained under standard conditions. All animal procedures were approved by the Institutional Animal Care and Use Committee of Yamaguchi University. The methods were carried out in accordance with the approved guidelines.

### Isolation and hypoxic preconditioning of PBMNCs

Mouse PBMNCs were isolated from peripheral blood by density gradient centrifugation using Lympholyte-Mammal (Cedarlane Laboratories) and suspended at a density of 2 × 10^6^ cells/ml in RPMI 1640 medium supplemented with 10% fetal bovine serum (FBS) and 1% penicillin-streptomycin. The hypoxic PBMNC culture was set at 2% O_2_ for 24 h (hypoxia), whereas the normoxic culture was set at 20% O_2_ for 24 h (normoxia), as described previously^12^.

### Injection of PBMNCs and apelin-13 into ischemic hindlimbs

To develop a mouse hindlimb ischemia model, the left femoral artery was ligated at the proximal portion, the saphenous artery was ligated at the distal portion, and all side branches were dissected free. Injection of PBMNCs and apelin-13 was performed in 40 mice immediately after surgery. Isolated mouse PBMNCs were cultured under hypoxic or normoxic conditions and intramuscularly injected into the ischemic hindlimbs at four time points (10 μl of PBS or 5 × 10^5 ^cells/point). Recombinant apelin-13 ([Pyr1]-apelin-13: 0.1 μmol·kg^−1^·day^−1^) (Bachem) or PBS was intraperitoneally injected for the initial seven days^38^,^39^. Mice were divided into five groups: (1) PBS injections intramuscularly and intraperitoneally (control, *n* = 8), (2) apelin-13 injections only (apelin, *n* = 8), (3) Normoxic PBMNC implantation with apelin-13 injections (N-PBMNC + apelin, *n* = 8), (4) Hypoxic PBMNC implantation (H-PBMNC, *n* = 8), and (5) Hypoxic PBMNC with apelin-13 injections (H-PBMNC + apelin, *n* = 8).

### Measurement of blood perfusion in ischemic hindlimbs

Blood perfusion of ischemic soles was measured before surgery and at post-operative days (POD) 0, 3, 7, 14, 21, and 28 using a laser speckle perfusion imaging system (OMEGA ZONE, Omega Wave). Both ischemic (left) and non-ischemic (right) soles were scanned simultaneously, and perfusion scores were obtained for each sole. The recovery rate of perfusion in ischemic hindlimbs was calculated by the ratio of ischemic to non-ischemic sole perfusion scores.

### Measurement of vascular reactivity

To evaluate arterial vasodilation in ischemic hindlimbs, acetylcholine chloride (Ach; 10 μmol/L) (Sigma Aldrich) was injected intravenously into mice with hindlimb ischemia, and the recovery rate of blood flow from baseline was calculated in both ischemic and non-ischemic soles at POD 28. To exclude the possibility of nitric oxide (NO)-independent perfusion after Ach infusion, *N*_ω_-Nitro-L-arginine methyl ester hydrochloride (L-NAME; 100 μmol/L) (Sigma Aldrich), an inhibitor of the vasodilation response to Ach^40^, was intravenously administered to ischemic mice 15 min before Ach infusion.

### Immunohistochemistry

At POD 28, five mice from each group were euthanized to collect quadriceps/adductor muscles from ischemic hindlimbs. Harvested muscles were embedded in OCT compound (Tissue-Tek; Sakura Finetek Japan) and snap-frozen in liquid nitrogen. Frozen muscle sections (5-μm thickness) were air-dried and fixed with 4% paraformaldehyde (PFA) for 20 min at room temperature. After blocking with a blocking reagent (DAKO) including 1% Triton X-100, sections were incubated at 4 °C overnight with DyLite488-conjugated *Lycopersicon esculentum* Lectin (1:100; DL-1174, Vector Laboratories) for endothelial cell labeling and Cy3-conjugated anti-α-SMA (1:100; C6198, Sigma-Aldrich) for mural cell labeling. Microvessels were evaluated under a fluorescence microscope (BZ-X700; Keyence). Twenty different fields from four different cross sections were randomly chosen, and the number of microvessels per square millimeter was counted using cell counting software (BZ-II analyzer, Keyence); the average microvessel diameter was also obtained using the same microscopic field.

### Femoral artery ligation model

The left femoral artery was ligated to induce ischemic arteries and all collateral branches were subsequently dissected free. Ischemic femoral and saphenous arteries were harvested from hindlimbs for quantitative RT-PCR (qPCR) at POD 0, 3, 7, 14, and 28 (*n* = 4). In addition, arteries and muscle tissues for immunohistochemistry were harvested at POD 3 (*n* = 3). APJ expression was evaluated by immunofluorescence using the following antibodies: rabbit polyclonal anti-APJ (1:200; ab84296, Abcam), mouse monoclonal anti-vWF (1:50;, M0616, DAKO), mouse monoclonal anti-α-SMA (1:50; ENZ-C34933, Enzo Life Sciences), FITC-conjugated goat anti-mouse IgG (1:500; F0479, DAKO), and Alexa Fluor 555-conjugated goat anti-rabbit IgG (1:500; A21430, Life Technologies).

### Western blot analysis

Western blot analysis was performed on proteins (10–30 μg) isolated from whole cell lysates, as described previously. The following primary antibodies were used: rabbit polyclonal anti-APJ (1:1000; ab84296, Abcam), rabbit monoclonal anti-PDGFR-β (1:10,000; ab32570, Abcam), and rabbit polyclonal anti-β-actin (1:5,000; NB600-532H, Novus Biologicals). Corresponding horseradish peroxidase-conjugated secondary antibodies (DAKO) were subsequently applied. Signals were visualized with an enhanced chemiluminescence western blot detection system (GE Healthcare UK Ltd).

### Immunocytochemistry

To evaluate APJ expression in PBMNCs, cells were plated onto fibronectin-coated cell culture plates and cultivated in 10% FBS/RPMI 1640 medium under normoxic or hypoxic conditions. Following fixation with 4% PFA, PBMNCs were incubated with anti-APJ antibodies (1:200; Abcam) followed by Alexa Fluor 488-conjugated goat anti-rabbit IgG secondary antibodies (1:500; A11008, Life Technologies). To examine APJ expression in vascular smooth muscle cells (VSMCs), mouse aortic smooth muscle cells (Applied StemCell Inc) were seeded onto non-coated 12-well culture plates (passages 6–9, 2.5 × 10^5 ^cells/well) and cultured for 24 h (*n* = 3). Following fixation with 4% PFA, cells were incubated with primary antibodies for α-SMA (Cy3-conjugated; 1:100; C6198, Sigma Aldrich) and APJ (1:200; ab84296, Abcam). For evaluation of PDGFR-β expression in VSMCs, VSMCs (2.5 × 10^5 ^cells/well) cultured with or without apelin-13 (1 μM) (*n* = 3) were incubated with primary antibodies against PDGFR-β (1:100; ab32570, Abcam) followed by Alexa Fluor 555-conjugated secondary antibodies. Positive cells were manually counted in five different randomly chosen fields per well under a fluorescence microscope.

### Quantitative RT-PCR analysis

Total RNA was extracted from ischemic arteries in the femoral artery ligation mouse model using an RNeasy Plus Mini Kit (Qiagen) and reverse transcribed using PrimeScript^®^ RT Master Mix (Perfect Real Time; Takara) following the manufacturers’ instructions. The cDNA templates were amplified using a QuantiTect SYBR Green PCR Kit (Qiagen). The primer sets were as follows: 5′-CCA CTGTGGGCCACTTATACC-3′ and 5′-CAGCCTTAGCCGAGCATTG-3′ (mouse *APJ*), 5′-TGGCAAAGTGGAGATTGTTGCC-3′ and 5′-AAGATGGTGATGGGCTTCCCG-3′ (mouse *GAPDH*). Quantitative RT-PCR analysis was performed using a Light Cycler instrument and associated software (Roche Applied Science, Japan); data were evaluated using the 2^−ΔΔCT^ method.

### Enzyme-linked immunosorbent assay (ELISA)

To assess the production of growth factors associated with neovessel maturation, the culture supernatant was collected from apelin-13-treated PBMNCs, and ELISA was performed to measure the production of PDGF-BB, TGF-β1, VEGF, HGF, and bFGF (R&D Systems) according to the manufacturer’s protocol. N-PBMNCs or H-PBMNCs (2 × 10^6 ^cells/ml) were plated onto 24-well plates and cultured with or without apelin-13 (1 μM) for 24 h (*n* = 4).

### Cell proliferation and migration assays

To evaluate the effect of apelin-13 on VSMC function, a proliferation assay was performed using the WST-8 protocol (Nacalai Tesque). VSMCs (1 × 10^4 ^cells/well) were plated onto a 96-well plate and incubated with apelin-13 (1 μM) or PDGF-BB (10 ng/ml; Sigma Aldrich) for 48 h (*n* = 6).

For the cell migration assay, transwell inserts with an 8.0-μm pore size (Corning Inc) were used. Human primary VSMCs (ATCC) were treated with apelin-13 (1 μM) for 24 h prior to re-plating onto a transwell insert. VSMCs (5 × 10^4 ^cells/ml) were suspended in 100 μl of culture medium and plated onto the insert, and 600 μl of culture medium with or without PDGF-BB (10 ng/ml) was added to the lower chamber (*n* = 3). After incubation for 12 h, the filters were fixed and stained with 0.5% crystal violet (Sigma Aldrich) in 20% methanol for 10 min. The number of cells that migrated through the filter pores was manually counted under a microscope. Five different randomly chosen fields from each filter were used for cell counting.

### Aortic ring assay

An aortic ring assay was performed according to a modified protocol^41^. Briefly, descending thoracic aortas were harvested from mice and placed in Opti-MEM^®^ (Life Technologies). The adventitia was dissected away and each aorta was cut into 0.5 mm rings under a microscope and cultured in Opti-MEM^®^ for 24 h. Next, aortic rings were embedded in 50 μl of growth factor-reduced Matrigel^®^ (BD biosciences) including N- or H-PBMNCs (2 × 10^6 ^cells/ml) in a 96-well plate supplemented with 150 μl of Opti-MEM^®^ with or without apelin-13 (1 μM) and cultured at 37 °C. The medium above the Matrigel^®^ was exchanged on days 3 and 5. On day 7, cultured aortic rings were fixed with 4% PFA and stained with an anti-α-SMA antibody (Cy3-conjugated; 1:100; C6198, Sigma-Aldrich). The number of sprouted vessels was counted under a light microscope, while α-SMA^+^ VSMCs surrounding sprouted vessels were evaluated using a fluorescence microscope.

### Statistical analysis

All data are expressed as mean ± SD. Differences between mean values of multiple groups were evaluated using a one-way ANOVA followed by a Bonferroni post-hoc test. Comparisons between two groups were performed using an unpaired Student’s *t*-test. Differences were considered significant at values of *p* < 0.05 or *p* < 0.01. All analyses were performed using STATA software (StataCorp).

## Additional Information

**How to cite this article**: Samura, M. *et al.* Combinatorial Treatment with Apelin-13 Enhances the Therapeutic Efficacy of a Preconditioned Cell-Based Therapy for Peripheral Ischemia. *Sci. Rep.* **6**, 19379; doi: 10.1038/srep19379 (2016).

## Supplementary Material

Supplementary Information
