# Supplementary material for: Combinatorial Treatment with Apelin-13 Enhances the Therapeutic Efficacy of a Preconditioned Cell-Based Therapy for Peripheral Ischemia
Source: Sci Rep. 2016 Jan 14;6:19379. doi: 10.1038/srep19379 (PMC4725909; doi:10.1038/srep19379)
Supplement: Supplementary Information [file srep19379-s1.pdf]

Supporting online materials for:

Combinatorial Treatment with Apelin-13 Enhances the Therapeutic Efficacy of a Preconditioned Cell-Based Therapy for Peripheral Ischemia

Makoto Samura, Noriyasu Morikage, Kotaro Suehiro, Yuya Tanaka, Tamami Nakamura, Arata Nishimoto, Koji Ueno, Tohru Hosoyama, Kimikazu Hamano

To whom correspondence should be addressed to:
Tohru Hosoyama (E-mail: toruhoso@yamaguchi-u.ac.jp)

This PDF file includes: Supplementary Figure S1.

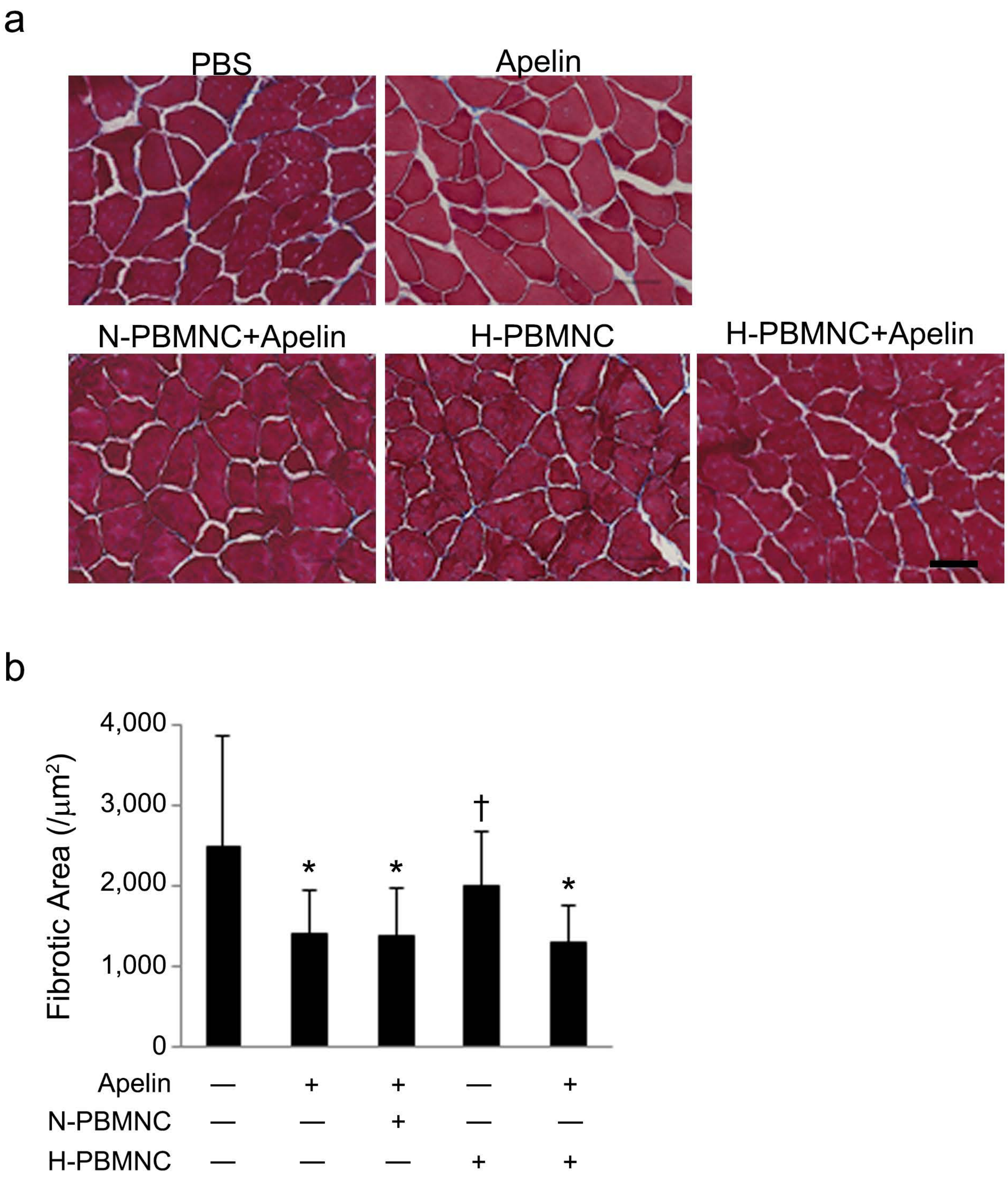

Supplementary Figure S1. Fibrosis analysis in ischemic hindlimbs on POD 28. (**a**) Masson's trichrome staining was performed on mouse muscle-cross sections of ischemic hindlimbs. Scale bar = 50 μm. Blue: fibrotic tissues. (**b**) Statistic analysis for fibrotic area in muscle-cross sections. *: $p < 0.01$ *vs*. PBS or H-PBMNC group; †: $p < 0.01$ *vs*. PBS group.
